# Shared care in the follow-up of early-stage melanoma: a qualitative study of Australian melanoma clinicians’ perspectives and models of care

**DOI:** 10.1186/1472-6963-12-468

**Published:** 2012-12-19

**Authors:** Lucie Rychetnik, Rachael L Morton, Kirsten McCaffery, John F Thompson, Scott W Menzies, Les Irwig

**Affiliations:** 1 Screening and Test Evaluation Program, School of Public Health, The University of Sydney, Sydney, Australia; 2 School of Public Health, The University of Sydney, Sydney, Australia; 3 Sydney Medical School, The University of Sydney, Sydney, Australia; 4 Melanoma Institute Australia (formerly Sydney Melanoma Unit), Sydney, Australia; 5 The Mater Hospital, North Sydney, Australia; 6 Royal Prince Alfred Hospital, Camperdown, Australia; 7 Sydney Melanoma Diagnostic Centre, Royal Prince Alfred Hospital, Sydney, Australia; 8 School of Public Health, The University of Sydney, Edward Ford Building (A27), Camperdown, NSW, 2006, Australia

**Keywords:** Melanoma, Follow-up, Shared care, Models of care

## Abstract

**Background:**

Patients with early stage melanoma have high survival rates but require long-term follow-up to detect recurrences and/or new primary tumours. Shared care between melanoma specialists and general practitioners is an increasingly important approach to meeting the needs of a growing population of melanoma survivors.

**Methods:**

In-depth qualitative study based on semi-structured interviews with 16 clinicians (surgical oncologists, dermatologists and melanoma unit GPs) who conduct post-treatment follow-up at two of Australia’s largest specialist referral melanoma treatment and diagnosis units. Interviews were recorded, transcribed and analysed to identify approaches to shared care in follow-up, variations in practice, and explanations of these.

**Results:**

Melanoma unit clinicians utilised shared care in the follow-up of patients with early stage melanoma. Schedules were determined by patients’ clinical risk profiles. Final arrangements for delivery of those schedules (by whom and where) were influenced by additional psychosocial, professional and organizational considerations. Four models of shared care were described: (a) surgical oncologist alternating with dermatologist (in-house or local to patient); (b) melanoma unit dermatologist and other local doctor (e.g. family physician); (c) surgical oncologist and local doctor; or (d) melanoma physician and local doctor.

**Conclusions:**

These models of shared care offer alternative solutions to managing the requirements for long-term follow-up of a growing number of patients with stage I/II melanoma, and warrant further comparative evaluation of outcomes in clinical trials, with detailed cost/benefit analyses.

## Background

Post-treatment follow-up is an important component of cancer care [1]. In many countries there is growing demand for oncology services and physician assistants and nurse practitioners have evolved in response to this demand [2-4]. There is also growing awareness of the important role of general practitioners, both in cancer management and in post-treatment follow-up [5-7]. A number of ‘shared care’ approaches have been described, including patients alternating follow-up visits between the oncologist and their local GP [8-10], or attending ‘specialist’ or ‘shared care’ GPs [11-13].

Melanoma is a growing burden worldwide and the fourth most common cancer in Australia [14-16]. Patients with AJCC stage I/II melanoma have high survival rates [17], but require long-term follow-up to detect recurrences and/or new primary tumours [18,19]. Melanoma patients also experience significant anxiety related to their disease [20]. Routine follow-up can provide reassurance but attending for follow-up can itself be a source of anxiety, and a burden in terms of time, travel and cost [21]. Sharing follow-up between melanoma specialists and local doctors is one solution to meeting the care needs of a growing population of melanoma survivors [22]. Some GPs may feel hesitant due to concerns about skills and capacity [23,24], but trials of GP-led care have been well received by GPs and patients in the UK [13,23,25-27]. In Australia, oncology specialists working in the field of breast cancer have also reported high levels of willingness to share care with other health professionals, but only 15% of their patients attended GPs for post-treatment follow-up [28]. The proportion of melanoma follow-up conducted as shared care is currently unknown. And while GP and patient experiences of shared care in melanoma follow-up have been described [26,27], no studies have examined shared care from the perspective of melanoma clinicians.

The purpose of this study was to examine specialist melanoma clinicians’ perspectives on the provision of post-treatment follow-up for patients with early stage melanoma in order to understand and inform future research on optimal models of care. In this paper we describe approaches to shared care in the follow-up of patients with AJCC stage I/II melanoma among melanoma specialists (surgical oncologists, dermatologists) and melanoma unit GPs (i.e. GPs based in a specialist melanoma unit and trained in melanoma follow-up); and outline four models of shared care as practiced in two of Australia’s largest tertiary referral melanoma diagnostic and treatment units. Melanoma specialists views on the overall functions of follow-up and follow-up intervals have been reported separately [29].

## Methods

This qualitative study was conducted in collaboration with two melanoma units in NSW, Australia. Melanoma Institute Australia (MIA) is one of the largest melanoma treatment units in the world and hosts the clinics of surgical oncologists and dermatologists, as well as melanoma unit GPs who conduct follow-up in some of its surgeons’ practices. The Sydney Melanoma Diagnostic Centre (SMDC) provides dermatology services and long-term monitoring of patients at high risk of primary melanoma, including those with previously treated disease. All clinicians at these units involved in post-treatment follow-up of patients with stage I/II melanoma were invited to participate in an in-depth, semi-structured interview about the nature and provision of follow-up care. All those invited (n=17) consented but one interview did not eventuate due to difficulties in finding a suitable time and 16 interviews were completed. The specialty and gender of participants are reported in Table 1. The study was approved by the Sydney South West Area Health Service Ethics Review Committee (Protocol No X09-0364).

**Table 1:** Specialty and gender of study participants

**Clinician characteristics (Total n= 16)**		
**Specialty**	Surgical Oncology	7
	Dermatology	5
	Primary Care, with focus on melanoma follow-up	4
**Gender**	Male	12
	Female	4

The interviews were conducted by three researchers (LR, RM or KM) and based on an interview schedule (Appendix 1). This schedule was developed following a systematic review of the literature [21] and consultations with melanoma clinicians. The interviews, each lasting 30–60 minutes, were conducted face-to-face (n=12) or by telephone (n=4) and all were recorded and transcribed. Analysis was conducted as a group process in which the researchers (LR, RM and KM) read all transcripts and independently prepared analytical notes that were discussed in regular meetings where the development of key categories was revised and refined [30,31]. The practice of shared care in melanoma follow-up was identified as an important category in the initial stages of analysis, and the commonalities and variations in practice, and potential explanations of these, were subsequently explored in the data [30,31]. Two participating clinicians were invited to provide feedback on the validity of these findings.

## Results

### Shared responsibilities in melanoma follow-up

Long term routine follow-up for stage I/II melanoma was often conducted as a form of shared care in which patients alternated between different clinicians at the melanoma units and/or their local / referring GP or skin cancer clinic.^a^ The melanoma unit clinicians noted a paucity of evidence on best practice in melanoma follow-up, and therefore follow-up schedules were primarily based on each patient’s expected risk of recurrence and of developing a new primary tumour, as well as on clinical guidelines [18]. A patient’s need for reassurance or further education to reinforce skin self-examination or sun-protective behaviours also influenced the recommended frequency of visits - especially in the first two years.

There was variation in whether, and for how long, surgical oncologists participated in long-term follow-up of patients with stage I/II melanoma; and for those who did, whether they themselves regularly conducted full body skin examinations as part of follow-up visits. Some surgical oncologists routinely referred patients with early stage melanoma to other clinicians for long-term follow-up e.g. to dermatologists at the melanoma unit (or if available one local to the patient) or to the patient’s GP or other referring doctor. Other surgical oncologists referred to one of several melanoma unit GPs, who are co-located in the melanoma unit facilities and to whom the surgical oncologists provide specialist support.

Surgical oncologists who preferred to retain greater, ongoing personal involvement in patients’ follow-up usually opted for shared care in the form of alternating visits with either a melanoma unit dermatologist or the patient’s local doctor. Although surgical oncologists preferred to focus on the wound site and detecting recurrences, they also described conducting full-body skin examinations if their patient had not received a comprehensive skin check elsewhere. Finally, surgical oncologists also described determining their own continued involvement in follow-up by a weighing patient’s clinical risks against logistic considerations. Thus they performed skin checks for patients who lived in remote country towns without access to a skin specialist, or organized follow-up with a local doctor for those with limited capacity for travel. The latter option was especially valued if the local doctor already had a good relationship with the melanoma unit and/or the patient and expressed interest in contributing to their follow-up.

The melanoma unit GPs and dermatologists identified long-term routine skin surveillance of patients with stage I/II melanoma as their primary responsibility, however they often also shared follow-up with other community-based doctors and two main approaches to shared care were identified. Some considered shared care as a way of providing for the psychosocial needs of anxious patients i.e. providing additional follow-up and reassurance for those who wanted more regular skin checks than was indicated by their risk profile. But others were more comfortable to alternate the clinically recommended schedule of skin checks with the patients’ local doctor. This partly depended on feedback from patients i.e. by instructing patients on what to look for, melanoma clinicians felt able to trust the capacity of many of their patients to make an assessment of skin examinations received elsewhere.

To conclude, recommended schedules for the follow-up of individual patients were primarily determined by their risk profile, but final arrangements for the delivery of those schedules (i.e. by whom and where) were influenced by many other considerations. Table 2 provides a summary of the multiple and competing variables that were reported by melanoma clinicians to influence the practice of shared care. The factors listed under ‘Continuing Care’ inclined melanoma specialists to either conduct follow-up themselves or refer to ‘in-house’ melanoma unit GPs. ‘Community Referral’ factors inclined melanoma unit clinicians to refer patients to attend follow-up with their local or referring doctor. In weighing factors in these two categories, the melanoma unit clinicians determined whether follow-up was best shared ‘in-house’ or with other clinicians – or indeed if and when patients were referred for sole follow-up with their local doctor.

**Table 2:** Factors considered in melanoma follow-up that determined the use of shared care-a summary of melanoma clinicians’ perspective

**‘CONTINUING CARE’ factors**	**Variables**	**‘COMMUNITY REFERRAL’ factors**
***Inclined melanoma unit clinicians towards specialist or ‘in-house’ follow-up e.g. by surgeon or melanoma unit dermatologist or melanoma GP***		***Inclined melanoma unit clinicians towards enabling follow-up by community doctors e.g. dermatologist, local GP or skin cancer clinic***
▪ Higher risk of recurrence or new primary disease (prior melanoma, tumor thickness, ulceration, mitotic rate, family history, skin type, number of moles etc)	**Clinical**	▪ Lower risk of recurrence or new primary disease
▪ Indications for extended post-surgical monitoring e.g. pain, hematomas, lymphodema, affected functioning		
▪ Patient request for ‘in-house’ follow-up by someone with identified melanoma expertise	**Patient Psychosocial**	▪ Proximity and travel to unit pose significant burdens; potential barrier for patient attending scheduled visits (live far away, have poor mobility etc)
▪ Patient allegiance to specialist with preference for attending with them personally		
		▪ Patient prefers follow-up with own family physician or local referring doctor, or happy to participate in shared care
▪ Patient very anxious; requires high emotional support and reassurance		▪ Patient organizes and coordinates follow-up with preferred providers and follow-up consistent with recommended schedule
▪ Patient uncomfortable with referral to local doctor for follow-up		
		▪ Patient knowledgeable, confident and conscientious in conducting skin self-examination
▪ Patient lackadaisical about skin surveillance and needs ongoing education and reinforcement of self examination		
▪ Patient lives close by or is able and willing to travel to unit for appointments		
▪ Emphasis on specialisation in follow-up; ie specialist training and/or location in melanoma unit to facilitate early detection of disease 1	**Melanoma Clinician**	▪ Professionally comfortable with sharing follow-up with non-specialist clinicians; especially when preferred by patient and/or addresses other psychosocial needs
▪ Sense of overall responsibility for ones patients; professional obligation to provide ongoing care or oversee quality of skin surveillance provided by others		▪ Sense of obligation to expand capacity of one’s practice to accommodate new melanoma patients
		▪ Value of health system efficiency and maximizing benefits for greatest number of patients i.e. focusing specialist care for those at greatest need / highest risk
▪ Value of knowing patient well and patient-doctor rapport to facilitate education, early diagnosis and treatment ie doctor is familiar with patients’ skin, character, lifestyle, preferences; and patient comfortable to ask questions or return if worried		
		▪ Value of efficient care for individual patients i.e. reducing burdens of travel and cost of follow-up relative to clinical returns for those with lowest risk of disease
▪ Clinical interest in observing surgical and clinical outcomes over the long-term; being able to personally monitor developments		
▪ Enjoyment of psychosocial aspects of follow-up ie regular contact with ‘well’ patients		
▪ Professional courtesy and goodwill towards referring doctor; inclined to offer continued contribution to follow-up even if specialist in-put not clinically necessary		
▪ Alternative follow-up with community doctor not available or accessible to patient	**Community Doctor**	▪ Local doctor perceived to be knowledgeable, skilled and competent in providing melanoma follow-up 1
▪ Local doctor’s skills and interest in follow-up unknown; specialist feels need to supervise follow-up more closely		
		▪ Local doctor known to melanoma unit; eg has other successful shared care arrangements with specialist clinicians
▪ Patient has no or poor relationship with local doctors		▪ Local doctor known to be interested and motivated to conduct melanoma follow-up
▪ Specialist or patient perceive local doctor not to have the knowledge, skills, capacity or interest to conduct melanoma follow-up		▪ Patient has established good and trusting relationship with local doctor
▪ Value of research roles and responsibilities of specialist unit; benefits of longitudinal data on patient outcomes	**Organizational (melanoma unit)**	▪ Limited capacity of specialist melanoma unit clinicians (surgical oncologists in particular) to provide long-term routine skin surveillance for patients at low risk of recurrence or new disease
▪ Institutional benefits of constituency and support-base for a specialist unit from maintaining ongoing relationships with current and past patients		

The important variables that impact on shared care are further sub-divided as follows: patient *clinical* and *psychosocial* variables; *melanoma clinician* variables; *community doctor* variables; and *organizational* (melanoma unit) variables. For example, patient characteristics included: patients’ overall risk of recurrence and new primary disease, patient preferences for follow-up, their level of anxiety and need for information and reassurance, and overall confidence in the alternative follow-up options. The melanoma clinician variables included different perspectives regarding their own and other clinicians’ roles and responsibilities in long-term melanoma follow-up. Community doctor variables related to the availability and reliability (sometimes based on feedback from patients) of the follow-up by the patient’s local or referring doctor. Finally, organizational factors were also identified by clinicians as important considerations in follow-up, including the finite capacity of a specialist melanoma unit to cope with ever-increasing numbers of patients requiring follow-up, and its additional functions in melanoma research (including accurate database maintenance).

### Four models of shared care

**Figure 1:**
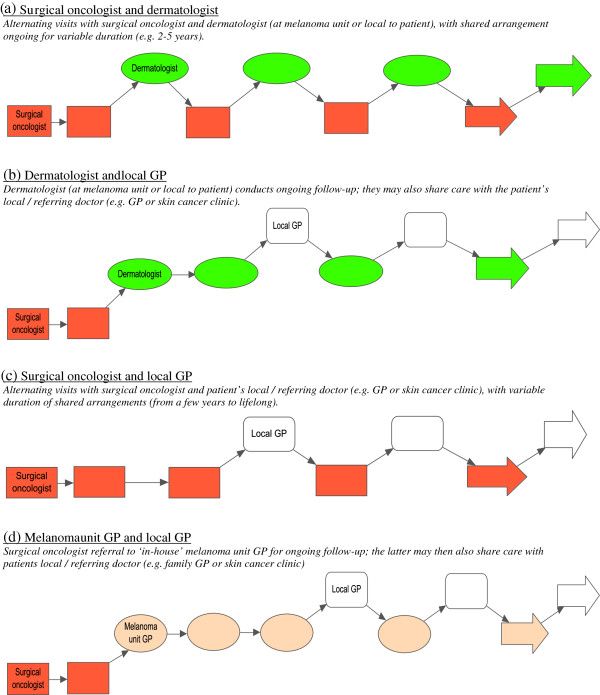
Four described models of shared care.

The four main models of shared care for early stage melanoma, as reported by the melanoma unit clinicians in this study, are summarized in Figure 1. These models were established through formal doctor-to-doctor referral or arranged by patients themselves. It is important to note that while the models reflect the most commonly discussed shared care arrangements, they do not reflect all possible variations of practice, and are not mutually exclusive. For example, shared care arrangements would sometimes change due to a patient’s altered clinical or social circumstances, or over time as the risk of recurrence decreased.

Model (a) comprised alternating visits with the surgical oncologist and a dermatologist, who was either based at the melanoma unit or local to the patient. This model of shared care could continue for different lengths of time (e.g. between 2 to 5 years post-treatment) after which on-going skin surveillance would be fully transferred to the dermatologist. Alternatively, model (b) comprised early handover by the surgical oncologists (e.g. after 1 or 2 visits) for ongoing post-treatment follow-up with a dermatologist. The dermatologist may then subsequently also share follow-up care with the patient’s own GP or skin cancer clinic who had originally referred the patient.

Model (c) comprised alternating visits with a surgical oncologist and the patient’s own local GP or skin cancer clinic. The duration of the shared care arrangements represented in model (c) was highly variable; some surgical oncologists reported alternating follow-up with the local doctor for a few years only post-treatment, but others described long-term or ongoing involvement in follow-up (e.g. for 10 or 15 years post-diagnosis), especially for those patients who specifically requested that they continue to see them.

Model (d) comprised early handover by the treating surgeon (e.g. after 1 or 2 visits) for ongoing post-treatment follow-up with one of the melanoma unit GPs. Sometimes the preferred arrangement was a slightly longer period of alternating visits between the two before the melanoma unit GP took over. For many patients, particularly those for whom travel was a significant burden, the melanoma unit GPs then also shared subsequent follow-up visits with the patient’s local /referring dermatologist, GP or skin cancer clinic. Model (d) enabled participating surgical oncologists and melanoma unit GPs to accommodate many of the ‘Continued Care’ factors in Table 2, but within a framework that relied on a less intensive use of specialists.

While model (a) was described as providing the highest level of specialist care, it was also recognized as highly resource intensive. The prolonged involvement of a surgical oncologist was also perceived by some melanoma unit clinicians as more than absolutely required for the majority of patients with stage I/II melanoma. As outlined in models (a) and (c), however, some surgical oncologists had ongoing involvement in follow-up, describing their sense of duty to continue to see patients they had treated if that was what those patients particularly requested. Shared care with local doctors relied on less intensive use of melanoma units, and for those living further away also reduced financial and travel burdens.

Model (d) required the least involvement of surgical oncology and dermatology specialists. Yet views on this arrangement varied, and a few specialists expressed uncertainty about the appropriateness of routine skin surveillance in a specialist tertiary referral unit being undertaken by the melanoma unit GPs, rather than only by surgical oncologists or dermatologists. Alternatively, those supporting model (d) said that because patients were being seen by GPs with an expressed interest and in-house training in melanoma follow-up, and with ready access to support from specialist colleagues, it provided better quality assurance for long term monitoring than referrals elsewhere. Model (d) was also recognized to provide accurate information for the unit’s database of patient records, which supports longitudinal melanoma research.

## Discussion

Australia has one of the highest rates of cutaneous melanoma in the world, with a growing population of patients requiring long-term post-treatment follow-up [14,18,32]. The majority of melanoma specialists are based in metropolitan areas and there is increasing demand for their services. This qualitative study offers valuable insights into the practice of shared care by melanoma unit clinicians, which is an important aspect of the provision of post-treatment follow-up for a growing population of melanoma survivors. As described in the study, melanoma specialists valued shared care in the follow-up of patients with stage I/II melanoma to accommodate the needs of their patients, and to manage the finite capacity of specialised units to provide routine skin surveillance. Patients’ clinical risk factors influenced follow-up options, as well as psychosocial and logistic considerations, such as patients’ anxiety or the distance between home and the melanoma unit and the patients’ capacity to travel. We describe the nature and range of factors that may be considered and weighed by melanoma specialists in their practice of shared care, and present four models of shared care from two of Australia’s largest melanoma units. It should be acknowledged however, that the perspectives reported in this study may differ from those clinicians who provide post-treatment melanoma follow-up in other settings, such as dermatologists not affiliated with melanoma units, GPs based in skin cancer clinics, or other GPs. Other melanoma units may also adopt alternative or additional models of shared care.

Many patients with AJCC stage I/II melanoma have a greater risk of developing a new primary melanoma than a recurrence [33]. In Australia the 10 year risk of mortality for melanomas with a Breslow thickness ≤1 mm is less than half the 10 year risk of developing a second primary melanoma, but for tumours with a Breslow thickness >1 mm the 10 year risk of mortality is substantially greater [34,35]. Although responsibility for detecting recurrence is not restricted to melanoma unit clinicians, improved treatment options may incline melanoma units more towards identifying options to provide continued ‘in-house’ follow-up for patients with thicker tumours. The described model (d) of specialists referring follow-up to melanoma unit GPs is a relatively new approach, which expands the capacity of a melanoma unit to provide follow-up by GPs who have greater access to specialist support than is available to most community GPs. Although this model does not overcome the burdens of time and travel that are reported by some patients as barriers to adherence with schedules [21], this was partly addressed by the melanoma unit GPs also sharing follow-up with other community-based doctors.

Patients also value GP participation in cancer follow-up, particularly for navigating the health system and providing information and psychosocial support [27,36,37]. Other models of shared care reported elsewhere have included community-based ‘shared care’ GPs who conduct follow-up for all cancers, including melanoma [13], and local GP-led melanoma follow-up supported by additional training and improved systems of referral to melanoma specialists when suspicious lesions are found [25]. All forms of shared care rely on clarity about roles and responsibilities of the clinicians, and these must also be understood by patients themselves [38]. Australian patient perspectives on the benefits and challenges of long term follow-up of stage I/II melanoma have also been reported [39]. Melanoma follow-up requires long-term commitment to regular skin surveillance and many patients may benefit from increased opportunities to plan in advance how this can be achieved. Explicit discussion of alternative options for post-treatment follow-up could become a formal part of patients’ considerations of treatment referral options. Printed patient information describing all available models for their follow-up, including who would conduct each aspect, qualifications and training of the practitioners, and any associated costs, could greatly assist this process.

## Conclusion

Melanoma clinicians consider many competing factors when they determine an appropriate model of follow-up care for their patients. A number of alternative models of shared care have been described, which rely on different levels of contribution from surgical oncologists, dermatologists and general practitioners. These models of shared care offer solutions to managing the requirements for long-term follow-up of a growing number of patients with AJCC stage I/II melanoma, and warrant further comparative evaluation of outcomes in clinical trials, with detailed cost/benefit analyses.

## Endnote

^a^Australia has a growing number of skin cancer clinics which are mostly staffed by general practitioners. They offer skin examinations and skin cancer diagnostic and treatment services, as well as referrals to specialists and specialist melanoma units

## Competing interests

The authors have no financial, commercial or other competing interests.

## Authors’ contributions

All authors contributed to the conception and design of the study and acquisition of the data. LR, RM and KM collaborated on the analysis and interpretation of data, with substantive contributions from LI, JT and SM. LR drafted and revised the manuscript, with all other authors providing critical feedback on important intellectual content resulting in substantial revisions. All authors read and approved the final manuscript.

## Appendix A. Clinician interview schedule

### A.1. Overall objectives of follow-up and clinician perspectives of patient needs

1. If we could start quite broadly, what would you say are your main aims in undertaking regular follow-up and monitoring for patients who have had a stage 1 or 2 primary melanoma?

2. What do you see as your main responsibility in relation to follow-up and monitoring for your melanoma patients?

3. What would you consider to be more within the scope / role / responsibility of other clinicians?

4. In terms of follow-up of people with stage 1 or 2 melanoma, what would you say are some of the similarities and differences between SMDC and the MIA?

### A.2. Clinicians own needs, experiences and motivations for follow-up

5. What about yourself – what would you say are some of the main benefits for you personally as a practitioner from having the opportunity to regularly see your follow-up patients?

6. How does it compare to some of the other clinical work that you do in terms of job satisfaction or enjoyment?

7. Do you ever worry about missing a melanoma?

8. Do you think there are any medico-legal implications?

### A.3. Patient experiences as perceived by clinicians

9. From your experience, what would you say are the main things the patients want from coming to see you for regular follow-up?

10. Do patients ever talk about how they felt before or after their appointment, how they are feeling while they are here?

### A.4. Factors influencing follow-up intervals and any implications for change

11. In terms of the time interval between appointments – how do you determine how often a patient should attend?

12. If there was good evidence that recurrence-detection rates and clinical outcomes were not affected by increasing the time interval between follow-up appointments (say from 6monthly to yearly) what other factors would you and your patients want to consider when reviewing intervals between appointments?

### A.5. Monitoring undertaken by others

13. Are there any aspects of follow-up care that are currently being done by others at SMDC or in the future could be done just as well or more efficiently for your patients?

14. Do you ever refer patients for follow up or skin checks with a local GP or a dermatologist other than at SMDC?

15. What are your thoughts on other possible benefits from following up patients over the long term, such as research benefits?

16. Do you refer any of your follow-up patients for skin photography?

### A.6. Patient education

17. Are you involved in follow-up patient education? What types of patient education do you do?

18. Is there anything else that you would like to see included in the follow-up or monitoring process?

## Pre-publication history

The pre-publication history for this paper can be accessed here:

http://www.biomedcentral.com/1472-6963/12/468/prepub
